# Cytokines and lymphocyte subsets are associated with disease severity of severe fever with thrombocytopenia syndrome

**DOI:** 10.1186/s12985-024-02403-0

**Published:** 2024-06-03

**Authors:** Li Song, Wenlu Zou, Gang Wang, Ling Qiu, Lintao Sai

**Affiliations:** 1 Department of Infectious Diseases, Qilu Hospital of Shandong University, Wenhua Xi Road 107, Jinan, Shandong 250012 China; 2 Department of Infectious Diseases, Shandong Public Health Clinical Center, Lieshishan Dong Road 11, Jinan, Shandong 250102 China

**Keywords:** Severe fever with thrombocytopenia syndrome, Severity, Cytokines, Lymphocyte subsets, Predictors

## Abstract

**Background:**

Severe fever with thrombocytopenia syndrome (SFTS) is an emerging infectious disease caused by severe fever with thrombocytopenia syndrome virus (SFTSV). Previous studies have indicated that SFTS patients have a high mortality rate, which may be related to cytokine storm and immune dysfunction. In our study, we analyzed differences in cytokines and lymphocyte subsets between severe and non-severe SFTS patients, with the aim of identifying predictors of severity.

**Methods:**

We retrospectively analyzed demographic characteristics, clinical data, cytokine profiles, and lymphocyte subsets from 96 laboratory confirmed SFTS patients between April 2021 and August 2023.

**Results:**

A total of 96 SFTS patients were enrolled, with a mean age of 65.05 (± 7.92) years old. According to our grouping criteria, 35 (36.5%) of these patients were classified as severe group, while 61 (63.5%) were classified as non-severe group. Univariate analysis revealed that age, interleukin-6 (IL-6), interleukin-8 (IL-8), interleukin-10 (IL-10), interferon-α (IFN-α), CD4 + T cell, and CD8 + T cell counts were risk predictors for the severity of SFTS. Further multivariable logistic regression analysis confirmed age, IL-6 levels, and CD4 + T cell counts as independent predictors of SFTS severity.

**Conclusions:**

Severe SFTS patients may experience cytokine storms and immune dysfunction. Aging, elevated levels of IL-6, and decreased CD4 + T cell count may serve as independent predictors for the severity of SFTS.

## Background

Severe fever with thrombocytopenia syndrome (SFTS), an emerging infectious disease caused by severe fever with thrombocytopenia syndrome virus (SFTSV), was first reported in China in 2010 [1]. The primary mode of transmission of SFTSV is through tick bites. However, person-to-person transmission has been identified through epidemiological investigations of SFTS patients [2]. SFTS patients typically present with fever, thrombocytopenia, leukopenia, and gastrointestinal symptoms. Some SFTS patients progress rapidly and develop multiple organ dysfunction syndrome (MODS), which is closely associated with fatal outcomes [1]. Case-fatality rates vary between 5% and 30%, and the average mortality rate remains high due to the lack of effective treatment [3].

At present, the pathophysiological mechanisms leading to severe illness in SFTS patients remain unclear. Previous studies have suggested that cytokine storms are among the pathophysiological features, with elevated levels of certain cytokines observed in severe SFTS patients [4–6]. Based on which, clinicians are exploring the use of biologics to treat SFTS and potentially reduce mortality rates [7]. In our study, we analyzed differences in cytokines and lymphocyte subsets between patients with non-severe SFTS and those with severe SFTS, aiming to identify predictors contributing to the severity of SFTS. We anticipate that our findings will aid in the early identification of severe SFTS cases and offer evidence to support clinicians in utilizing biologics to mitigate cytokine storms.

## Methods

### Study design

To investigate the roles of cytokines and lymphocyte subsets in the severity of SFTS, we conducted a retrospective analysis of demographic characteristics, clinical data, cytokines, and lymphocyte subsets from 96 laboratory confirmed SFTS patients. The patients were treated at two tertiary hospitals (Qilu Hospital of Shandong University and Shandong Public Health Clinical Center) in Shandong province between April 2021 and August 2023. This study was approved by the Medical Ethical Committee at Qilu Hospital of Shandong University (2021 − 120), and written informed consent was obtained from all enrolled patients or their guardians. All methods were performed in accordance with the Declaration of Helsinki and relevant guidelines and regulations.

### Patient enrollment and grouping

Patients were diagnosed with SFTS and enrolled based on the following criteria: (i) clinical presentation featuring acute fever and thrombocytopenia; (ii) serum testing positive for SFTSV RNA detected by real-time polymerase chain reaction (RT-PCR) assay. Subsequently, enrolled SFTS patients were further categorized into the severe and non-severe groups. Patients were classified as severe if they presented with dysfunction in three or more organ systems, meeting the following criteria: (1) secondary pulmonary infection requiring high-flow oxygen therapy or ventilator-assisted ventilation; (2) secondary bloodstream infection leading to septic shock necessitating vasopressor therapy; (3) hepatic dysfunction: serum bilirubin ≥ 2–3 mg/dL or liver function tests showing values ≥ twice normal; (4) acute kidney injury: oliguria ≤ 400 mL/24 hours or increasing creatinine (≥ 2–3 mg/dL); (5) hemorrhagic tendency: activated partial thromboplastin time (APTT) increase > 25% or platelet counts ≤ 30*10^9/L; (6) neuropsychiatric symptoms; and (7) heart failure.

### Data collection

The relevant data of enrolled SFTS patients, including demographic characteristics, clinical data, and immune parameters, were collected and organized based on their electronic medical records. Immunological parameters were evaluated through changes in cytokine levels and lymphocyte subsets, which were collected and analyzed within 7 days after the onset.

### Measurement of immunological parameters

Secondary bacterial infections may lead to elevated immune parameters, which could potentially introduce bias to the results. Therefore, we excluded cases with concurrent infections when collecting peripheral blood specimens. Peripheral blood samples coated with ethylenediaminetetraacetic acid (EDTA) were collected from SFTS patients within 7 days after the onset. Plasma was separated by centrifugation at 3000 rpm for 20 min and stored at -80 °C for subsequent analysis. According to the manufacturer’s instructions, serum levels of interleukin-6 (IL-6), interleukin-8 (IL-8), interleukin-10 (IL-10), interferon-α (IFN-α), and interferon-γ (IFN-γ) were measured by immunofluorescence using a cytokine combination assay kit (Saiji, Nanchang, China).

Peripheral blood samples treated with EDTA were stained using mouse anti-human monoclonal antibodies following the manufacturer’s instructions. Subsequently, white blood cells were washed and resuspended in phosphate-buffered saline (PBS), and then analyzed for CD4 + and CD8 + T lymphocytes using BD FACSCanto flow cytometer (BD, Franklin Lakes, USA).

### Statistical analysis

Statistical analysis was conducted using SPSS software (version 26.0). Categorical variables were presented as rates and compared by χ^2^ test or Fisher’s exact test. Continuous variables were expressed as mean ± SD (standard deviation) or median (IQR, interquartile range) and compared using the Mann–Whitney U test or Student’s t test. Normality and homogeneity of variance tests were conducted for continuous variables. If the data did not follow a normal distribution, the Mann-Whitney U test was applied for comparison. Conversely, if both groups exhibited a normal distribution and equal variances, Student’s t-test was used for comparison. Variables with a *p*-value < 0.05 in univariate analysis were further assessed using multivariable logistic regression analysis to identify independent risk factors for severe SFTS patients. Receiver operating characteristic (ROC) curve analysis was employed to evaluate the sensitivity and specificity of independent risk factors in predicting the severity of SFTS. A *p*-value < 0.05 was considered statistically significant for all analyses.

## Results

### Demographic characteristics and clinical data

A total of 96 laboratory confirmed SFTS patients were enrolled in the study. Among them, 35 (36.5%) patients, with a mean age of 68.77 (± 7.08) years, were classified into the severe group, while 61 (63.5%) patients, with a mean age of 62.92 (± 7.63) years, were categorized into the non-severe group. The difference in age between the two groups was statistically significant (*P* < 0.001). However, the gender distribution between the two groups showed no statistical significance. The presence of clinical data, including diabetes, hypertension, and coronary disease, was compared between the two groups, yielding no statistical significance (*P* = 0.062, *P* = 0.253, and *P* = 1.000, respectively). The demographic characteristics and clinical data of these patients are summarized in Table 1.

**Table 1:** Characteristics of demography, clinical data, cytokines and lymphocyte subsets of the patients with SFTS

		Total (*n* = 96)	Non-severe group (*n* = 61, m = 0)	Severe group (*n* = 35, m = 14)	*P* Value
Age (years), mean ± SD		65.05 ± 7.92	62.92 ± 7.63	68.77 ± 7.08	< 0.001
Gender	Male (%)	46 (47.9%)	28 (45.9%)	22 (62.9%)	0.109
	Female (%)	50 (52.1%)	33 (54.1%)	13 (37.1%)	
Diabetes (%)		13 (13.5%)	6 (9.8%)	7 (20.0%)	0.161
Hypertension (%)		23 (24.0%)	13 (21.3%)	10 (28.6%)	0.422
Coronary disease (%)		6 (6.3%)	4 (6.6%)	2 (5.7%)	0.870
Immunological parameters					
IL-6 (pg/ml), median (IQR)		9.59 (3.28–33.68)	5.40 (2.50-13.94)	30.00 (10.16–71.81)	< 0.001
IL-8 (pg/ml), median (IQR)		4.85 (1.16–19.14)	3.20 (1.03–9.56)	15.44 (3.28–40.10)	0.002
IL-10 (pg/ml), median (IQR)		5.19 (2.03–17.86)	2.83 (1.27–9.93)	14.50 (5.64–44.25)	< 0.001
IFN-α (pg/ml), median (IQR)		4.27 (0.63–67.31)	2.50 (0.27–24.73)	24.81 (2.50-175.40)	0.006
IFN-γ (pg/ml), median (IQR)		13.54 (2.50-97.24)	14.99 (2.50-89.74)	12.09 (1.69-211.47)	0.867
CD4^+^ cell counts/µl, median (IQR)		250 (120–467)	338 (148–552)	160 (75–251)	< 0.001
CD8^+^ cell counts/µl, median (IQR)		253 (118–460)	300 (140–546)	160 (91–305)	0.016

Abbreviations: IQR, interquartile range; SD, standard deviation; n: number of cases; m: number of deaths

### Immunological parameters

Immunological parameters, including cytokines and lymphocyte subsets, were compared (Table 1). The serum levels of IL-6, IL-8, IL-10, and IFN-α in the severe group were significantly higher than those in the non-severe group, with significant differences observed between the two groups. Although the level of IFN-γ increased in both groups, there was no significant difference noted between them. Additionally, the counts of CD4 + and CD8 + T cells in the severe group were significantly lower than those in the non-severe group, with statistically significant differences observed between the two groups.

### Multivariable logistic regression analysis

As shown in Table 1, seven variables exhibited statistical significance between the two groups. To further determine the impact of each statistically significant variable on the severity of SFTS, these variables underwent multivariable logistic regression analysis. The results indicated age (OR: 1.113; 95%CI: 1.026–1.206; *P* = 0.010), the level of IL-6 (OR: 1.013; 95%CI: 1.002–1.025; *P* = 0.023) and the count of CD4^+^ T cells (OR: 0.995; 95%CI: 0.990–0.999; *P* = 0.025) were independently associated with the severity of SFTS (Table 2).

**Table 2:** Multivariable logistic regression analysis on statistically significant variables

Variables	B	SE	Wals	*P* value	OR	95% CI
Age (years)	0.107	0.041	6.725	0.010	1.113	1.026–1.206
IL-6 (pg/ml)	0.013	0.006	5.163	0.023	1.013	1.002–1.025
IL-8 (pg/ml)	0.000	0.002	0.028	0.868	1.000	0.995–1.004
IL-10 (pg/ml)	0.012	0.010	1.461	0.227	1.012	0.993–1.032
IFN-α (pg/ml)	0.000	0.000	0.332	0.565	1.000	0.999–1.001
CD4^+^ T cell (counts/µl)	-0.005	0.002	5.053	0.025	0.995	0.990–0.999
CD8^+^ T cell (counts/µl)	0.002	0.001	3.447	0.063	1.002	1.000-1.005

### Receiver operating characteristic curve analysis

**Fig. 1:**
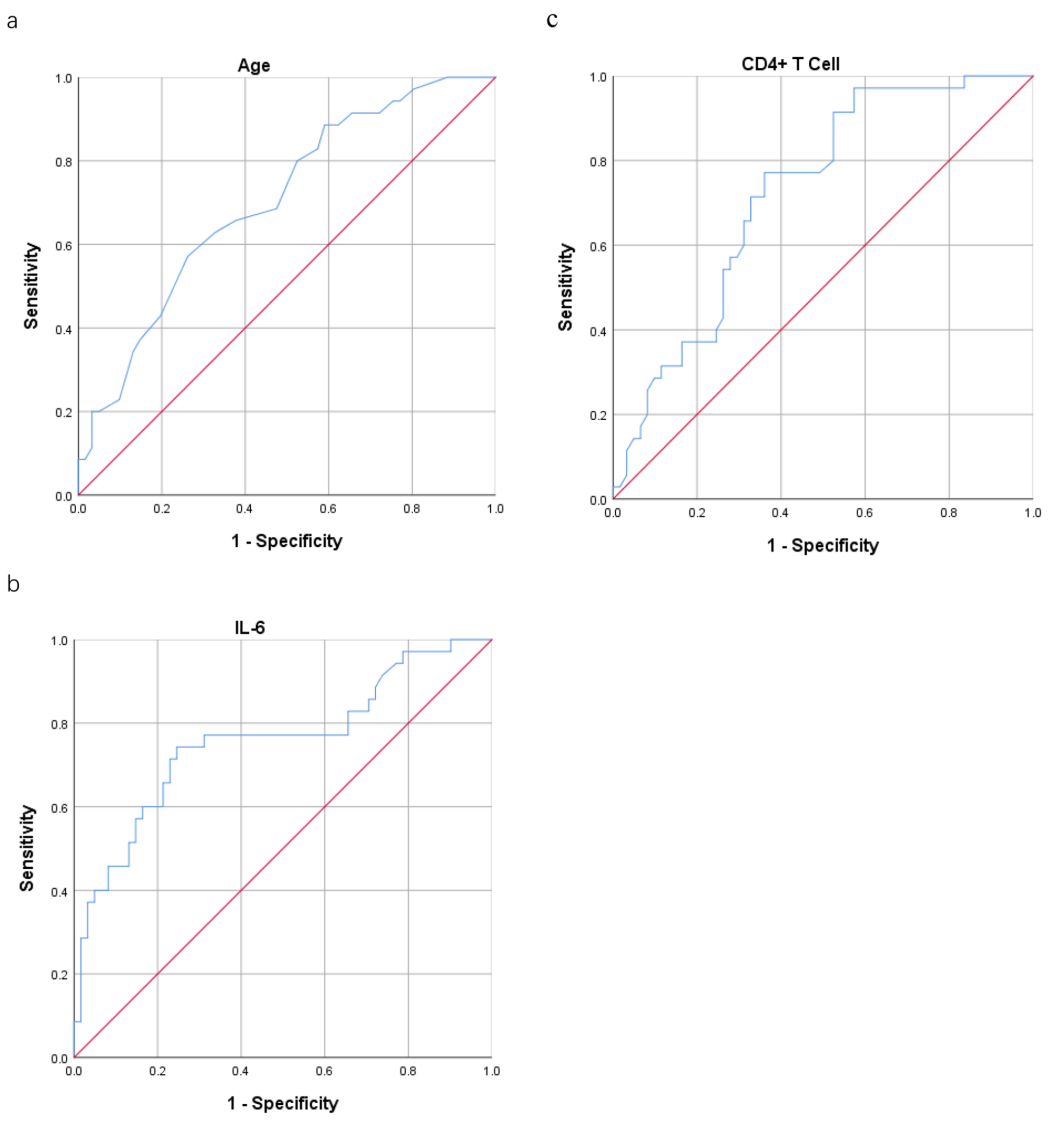
Receiver operating characteristic (ROC) curve analysis of independent predictors for severe patients with SFTS. **(a)** ROC curve of age (Cutoff value: 67.5; Area under curve: 0.704; 95%CI: 0.598–0.809; Sensitivity: 60.0%; Specificity: 70.0%; *P* < 0.001); **(b)** ROC curve of IL-6 level (Cutoff value: 13.8; Area under curve: 0.757; 95%CI: 0.650–0.864; Sensitivity: 74.0%; Specificity: 75.0%; *P* < 0.001); **(c)** ROC curve of CD4^+^ T cell count (Cutoff value: 255; Area under curve: 0.723; 95%CI: 0.623–0.824; Sensitivity: 77.0%; Specificity: 64.0%; *P* < 0.001)

Receiver operator characteristic (ROC) curve analysis was used to evaluate the sensitivity and specificity of each independent predictor in predicting the severity of SFTS (Fig. 1). The cut-off value of age to predict the severity of SFTS was determined to be 67.5 years old, with a sensitivity of 60.0% and specificity of 70.0%. For the IL-6 level, the cut-off value to predict severity was 13.8 pg/ml, yielding a sensitivity of 74.0% and specificity of 75.0%. Similarly, the cut-off value of CD4 + T cell count to predict severity of SFTS was determined to be 255 cells/µl, with a sensitivity of 77.0% and specificity of 64.0%.

## Discussion

High mortality of SFTS may be associated with cytokine storm and immune dysfunction. Therefore, we compared cytokines and lymphocyte subsets between severe and non-severe SFTS patients to identify predictors for severe patients.

Interleukin-6 (IL-6) is a pleiotropic cytokine [8], known for its role in inducing the acute immune response to combat infectious agents and facilitate tissue repair [9]. However, while IL-6 expression is tightly regulated, excessive release can contribute to the development of inflammatory cytokine storms and severe inflammatory diseases [10]. A previous study has identified IL-6 as a promising biomarker for assessing severity and as a prognostic indicator during cytokine storms [11]. In our study, we observed that the levels of IL-6 were higher in the severe group compared to the non-severe group. Furthermore, the results of multivariable logistic regression analysis suggested that IL-6 might serve as an independent predictor for severity in SFTS.

Interleukin-8 (IL-8), produced by various cell types, serves as a potent neutrophil chemotactic agent and activator [12]. In the context of local inflammation, IL-8 facilitates neutrophil migration, enhances vascular permeability, and promotes angiogenesis [13]. Previous studies have highlighted elevated IL-8 secretion levels in deceased SFTS patients [6, 14]. In our research, we similarly observed higher levels of IL-8 in severe SFTS patients, with a statistically significant difference between the two groups. However, despite this association, our analysis did not identify an elevated level of IL-8 as an independent risk factor for the severity of SFTS.

Interleukin-10 (IL-10) is a cytokine known for its anti-inflammatory properties, crucial for immunoregulation during infection. It plays a vital role in limiting excessive host damage caused by the immune reactions and in controlling the immune response to pathogens [15]. However, in certain infections, such as viral, fungal, helminth, or bacterial infections, excessive secretion of IL-10 may hinder pathogen clearance and contribute to the development of chronic disease [16, 17]. Although IL-10 was not identified as an independent predictor for the severity of SFTS in our analysis, we observed a statistically significant difference in IL-10 levels between the severe and non-severe groups, consistent with previous reports [6]. We speculate that the significantly elevated levels of IL-10 might contribute to immune dysregulation, potentially leading to the progression of SFTS [18, 19].

Interferons (IFNs), including type I (IFN-α and IFN-β), type II (IFN-γ), and type III (IFN-λ), play a crucial role in initiating effective antiviral immune response by disrupting virus replication within host cells, thereby promoting antiviral immunity [20]. Previous research on cytokines in SFTS has indicated elevated levels of IFN-α among deceased patients [4, 21]. In our study, we similarly observed higher levels of IFN-α in the severe group, with a statistically significant difference between the severe and non-severe groups. While some studies have reported significantly increased levels of IFN-γ in deceased SFTS patients [4, 21, 22], others have found lower levels of IFN-γ in SFTS patients compared to healthy individuals [5]. However, our study did not reveal a significant difference in IFN-γ levels between the severe and non-severe groups. These discrepancies in findings may be attributed to differences in the populations being studied.

Most SFTS patients are usually elderly, and our study confirms previous findings that age is an independent risk factor for the severity of the disease [14]. Immune function generally decreases with age, and impaired immune function can accelerate disease progression [23]. In our previous study, we observed decreased counts of CD4 + and CD8 + T cells in SFTS patients with invasive pulmonary aspergillosis [24]. Similarly, in the present study, we found decreased counts of CD4 + and CD8 + T cells in the severe group. Notably, CD4 + T cell count emerged as an independent predictor for the severity of SFTS. According to the ROC curve analysis, the sensitivity and specificity for predicting the severity of SFTS were higher when the count of CD4 + T cells was below 255/µl. In severe SFTS cases, the reduction in CD4 + and CD8 + T cell counts results in an insufficient number of active T cells to engage in cellular immune responses, thereby compromising cellular immune function and increasing the risk of infection from other pathogens. Therefore, the severity of SFTS may indeed be associated with the decreased counts of CD4 + and CD8 + T cells.

## Conclusion

In summary, the present study confirmed that ageing, elevated IL-6 level and reduced CD4 + T cell counts may serve as independent predictors for the severity of SFTS. Identifying these predictors could aid in assessing the severity of SFTS and implementing earlier interventions, potentially improving outcomes for severe SFTS patients.

## Data Availability

No datasets were generated or analysed during the current study. The data used and analyzed during the current study are available from the corresponding author on reasonable request.
